# Aberrant sense of agency induced by delayed prediction signals in schizophrenia: a computational modeling study

**DOI:** 10.1038/s41537-023-00403-7

**Published:** 2023-10-16

**Authors:** Tsukasa Okimura, Takaki Maeda, Masaru Mimura, Yuichi Yamashita

**Affiliations:** 1 Department of Neuropsychiatry, Keio University School of Medicine, Tokyo, Japan; 2 Medical Institute of Developmental Disabilities Research, Showa University, Tokyo, Japan; 3 Department of Psychiatry, Sakuragaoka Memorial Hospital, Tokyo, Japan; 4 Center for Preventive Medicine, Keio University, Tokyo, Japan; 5 Department of Information Medicine, National Institute of Neuroscience, National Center of Neurology and Psychiatry, Tokyo, Japan

**Keywords:** Health sciences, Schizophrenia, Neural circuits

## Abstract

Aberrant sense of agency (SoA, a feeling of control over one’s own actions and their subsequent events) has been considered key to understanding the pathology of schizophrenia. Behavioral studies have demonstrated that a bidirectional (i.e., excessive and diminished) SoA is observed in schizophrenia. Several neurophysiological and theoretical studies have suggested that aberrancy may be due to temporal delays (TDs) in sensory-motor prediction signals. Here, we examined this hypothesis via computational modeling using a recurrent neural network (RNN) expressing the sensory-motor prediction process. The proposed model successfully reproduced the behavioral features of SoA in healthy controls. In addition, simulation of delayed prediction signals reproduced the bidirectional schizophrenia-pattern SoA, whereas three control experiments (random noise addition, TDs in outputs, and TDs in inputs) demonstrated no schizophrenia-pattern SoA. These results support the TD hypothesis and provide a mechanistic understanding of the pathology underlying aberrant SoA in schizophrenia.

## Introduction

Schizophrenia is a common and disabling mental disorder^1^. The pathology of schizophrenia remains unclear, and effective treatments have not been developed^2^. Self-disturbance is a core feature of the illness^3^. Recently, this symptom has been studied in empirical research from the viewpoint of an aberrant sense of agency (SoA)^4–7^. SoA is the feeling that a person is causing and controlling their own actions and their effect on the world^8^. A dynamic combination of two key mechanisms is assumed to underlie SoA^5,9^. One is a predictive component; according to the classical comparator model of SoA^8,10^, comparison between the predictions of sensory consequences and actual sensory feedback provides the basis for SoA. If the comparison matches, sensory events are recognized as self-generated and SoA ensues. If there is a mismatch, SoA diminishes. The second component is retrospective, in which SoA is retrospectively generated based on inference (deducing causality based on contextual information about the causes of events)^5,9^.

**Fig. 1:**
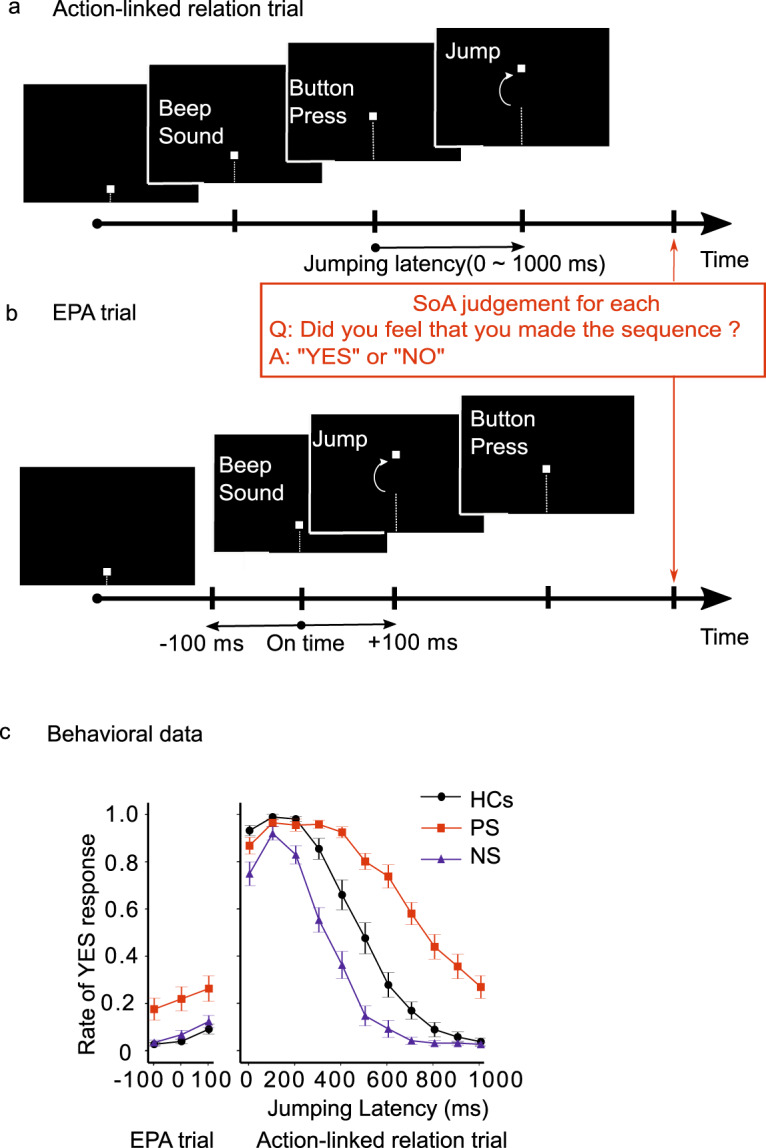
Sense of agency (SoA) task and behavioral data^6,7^. **a** The task referred to as an ‘action-linked relation’ trial in an agency-attribution task as a quantitative measure of SoA. **b** The task referred to as an event prior to action ‘EPA’ trial. **c** The mean rate of “yes” responses for SoA judgement among 35 healthy controls (HC)s, 30 paranoid-type schizophrenia (PS) subjects, and 20 negative symptom-predominant schizophrenia (NS) subjects. Bars represent standard error.

One representative task to measure SoA is the Keio method developed by Maeda et al. ^6,7^ (Fig. 1a, b). The task is an explicit measure of SoA in which subjects answer ‘yes’ or ‘no’ about SoA judgments. Maeda et al. ^6,7^ designed a task evaluating experiences of temporal causal relations between an intentional action and an external event. Moreover, Maeda et al. ^6,7^ demonstrated that patients with paranoid-type schizophrenia (PS) showed excessive SoA, whereas patients with negative symptom-predominant schizophrenia (NS) showed diminished SoA compared to healthy controls (HCs) (Fig. 1c). In addition, using a modified version of the SoA task (Keio method), Koreki et al. ^11^ suggested that altered SoA in patients with schizophrenia may be related to delayed prediction because of the impaired predictive component.

While the neural substrate of aberrant SoA in schizophrenia remains unclear^12^, an increasing number of studies show the neural substrates of malfunctioning predictive signals and efference copy in schizophrenia^13–22^. For example, using event-related potentials (ERPs) to an auditory stimulus, Whitford et al. ^17^ demonstrated that N1 suppression to the stimulus elicited by a self-generated action was reduced in schizophrenia. Moreover, this reduced N1 suppression can be ameliorated by delivering an auditory stimulus with a 50 ms delay. Changes in N1 suppression were also correlated with fractional anisotropy (FA) of the arcuate fasciculus in diffusion tensor imaging (DTI)^17^. Subsequent studies have shown that, in schizophrenia patients with delusions of control, the FA in the cingulum bundle is lower than that in patients with no history of delusions or in HCs, suggesting that structural damage to the cingulum bundle could lead to aberrant prediction error signals during self-generated actions, resulting in the development of delusions of control^19^. Similarly, the reduced magnetization transfer ratio of the cingulum bundle has been shown to be correlated with severity of delusional control and passivity symptoms^22^.

Based on these findings, it is reasonable to hypothesize that a temporal delay in signal transduction of efference copy may cause SoA to malfunction. In the current study, we examined this hypothesis using a computational modeling approach, reproducing behavioral features of aberrant SoA in patients with schizophrenia^6,7^ with a recurrent neural network (RNN) model (Supplementary Fig. 1; see Methods).

**Fig. 2:**
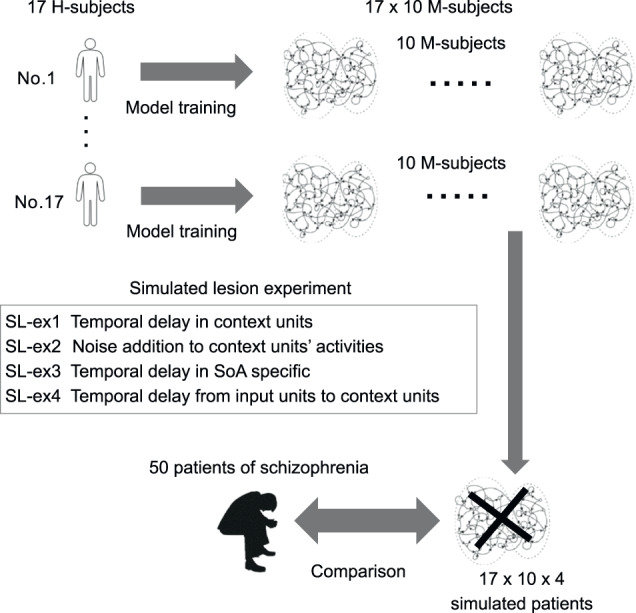
Overview of experimental procedure. The behavioral data of 17 healthy controls (H-subjects)^6,7^ were used as training data. Ten recurrent neural network (RNN) models, referred to as model subejcts (M-subjects), were generated for each H-subject. After we checked whether each M-subject successfully reproduced the behavioral features of the SoA task in the corresponding H-subject test data, four lesion experiments were simulated through M-subjects, including the hypothesis in the current study. We evaluated the similarity in SoA judgment between behavioral data in patients with schizophrenia and the lesioned M-subjects.

**Fig. 3:**
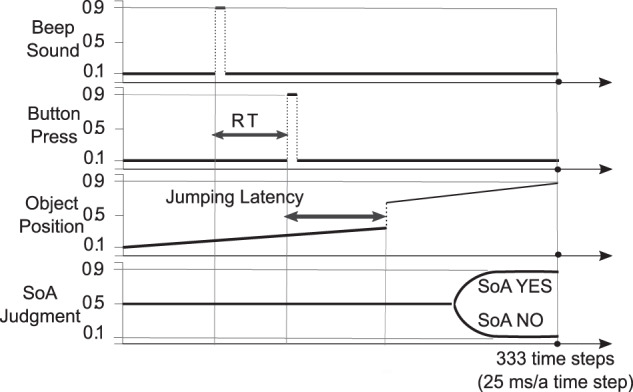
Sensorimotor sequences encoding the SoA task trial. Beep sound (first row), button press (second row), object position, (third row), SoA judgment (fourth row) are shown. Values for each sequence were mapped to the range of 0.1–0.9. If there was a beep sound, then the value = 0.9, and if not, 0.1. If the button was pressed, then the value = 0.9, and if not, 0.1. Values for object position started at 0.1 at the start of a trial and increased to 0.9 by the end of the trial. Values for SoA judgment started at 0.5 at the start of a trial and increased to 0.9 by the end of the trial in case of SoA YES, and 0.1 in case of SoA NO.

The experimental procedure is illustrated in Fig. 2 (see Methods). We trained RNN models against the actual behavioral data of 17 HCs (referred to as H-subjects) which were acquired in a previous study^6,7^. The sensory inputs to the RNN model were visual (object position), auditory (beep sound), and proprioceptive (button press). The outputs were sensory input predictions and judgment regarding SoA. An example of a trial sequence used in the simulation is shown in Fig. 3. Additionally, there were ten context units with recurrent inputs to every context unit (see Methods). The trained RNN models were treated as simulated subjects in the experiments (referred to as M-subjects). We generated 10 M-subjects from each H-subject, generating 170 M-subjects in total. After training the M-subjects, we checked whether each M-subject successfully reproduced the behavioral features of the SoA task in the corresponding H-subject (details in the Methods). To examine the hypothesis that temporal delays (TDs) in predictive signal transduction cause aberrant SoA in schizophrenia, we conducted a simulated lesion experiment with M-subjects (SL-ex1). We conducted three additional lesion experiments (SL-ex2 to 4) as the control experiments for the hypothesis we proposed, and compared actual behavioral data^6,7^ in patients with schizophrenia and that in M-subjects simulated by the four experiments.

The proposed RNN model successfully reproduced the behavioral features of HCs in the SoA task. In addition, a schizophrenia-pattern aberrant SoA was reproduced in the lesion experiment by adding a TD in signal transfer to the predictive processing network. A schizophrenia-pattern aberrant SoA was not observed in the three other lesion experiments. These results support the hypothesis that a TD in predictive signal transduction causes a malfunctioning SoA in patients with schizophrenia.

The approach we have used in this study is one of computational psychiatry, an emerging field that will help to bridge the explanatory gap between abnormalities observed at neurological and behavioral levels in mental disorders^23–29^. Specifically, RNN models can simulate behaviors arising from the dynamics of neural activities and are useful for extracting the characteristic features of neural systems. The present study could provide a mechanistic explanation that bridges the gap between aberrant SoA in schizophrenia and the neurophysiological as well as neuroimaging findings.

## Results

### Reproducing behavioral features of the SoA task in healthy participants

**Fig. 4:**
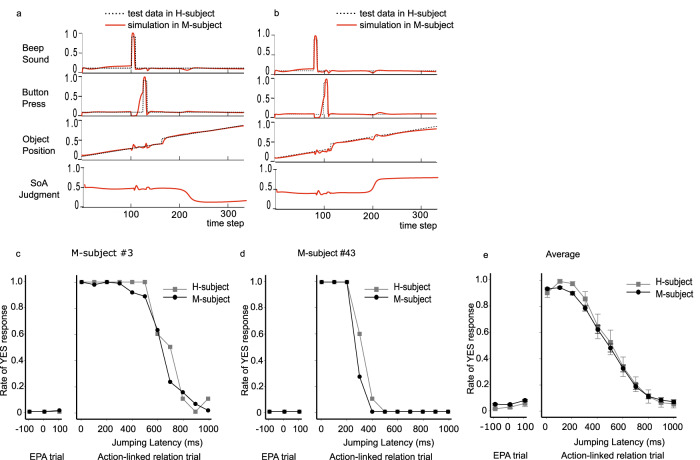
Comparison between test data in H-subjects and simulations in M-subjects. Two examples of this comparison at the trial sequence level (**a**, **b**). Two examples of this comparison at the subject level (**c**, **d**). **e** The comparison at the group level. Bars represent the standard error.

The RNN model successfully reproduced the behavioral features of HCs in the SoA task. Figure 4a, b illustrates examples of single trial-level reproduction of sensorimotor sequences during SoA task execution with two different jumping latency conditions. Figure 4c, d illustrates two examples of subject-level reproduction of SoA judgments in two M-subjects, where the SoA task was summarized as a plot of the rate of ‘yes’ responses for each jumping latency. The proposed model successfully reproduced characteristic features, including the inflection point of the sigmoidal yes-rate curve for each H-subject. As a result, the proposed model successfully reproduced the group-level performance of the SoA task (Fig. 4e). The average SoA judgments for the 170 M-subjects almost perfectly matched the average SoA judgments for the 17 H-subjects.

### SL-ex1 (TD in context)

**Fig. 5:**
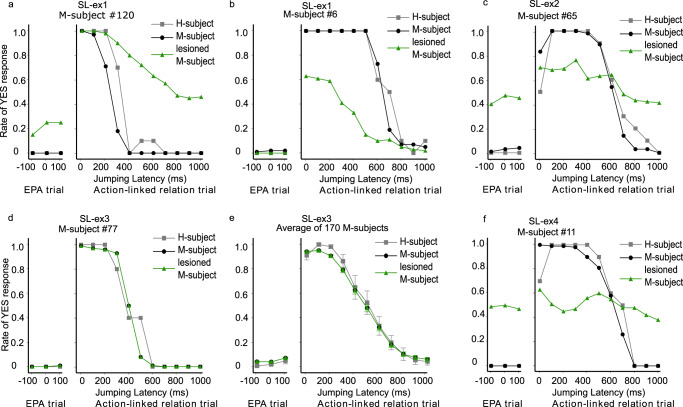
Examples of the results in the lesion experiments. The lesion experiment, SL-ex1 (TD in context), induced two different (i.e., excessive and diminished) types of changes in sense of SoA judgment. An example of the excessive-type change (**a**). An example of the diminished-type change (**b**). **c** Example of the results in SL-ex2 (random noise). The yes-rates with regard to SoA in both short and long conditions of jump delay were changed into 0.5, i.e., like random SoA judgment. **d**, **e** Comparisons of sense of agency between M-subjects in SL-ex3 (TD in output) and in H-subjects. The data for M-subject No. 77, trained using H-subject No. 8 (**d**). The average rate for 170 M-subjects (**e**). Bars represent the standard error. In (**d** and **e**), although three lines were plotted, only two lines (gray and green) were seen, since the black and green lines were fully identical. **f** An example of results in SL-ex4 (TD in input). The ‘yes’ rates with regard to SoA in both short and long jump delay conditions were changed to 0.5, i.e., akin to a random SoA judgment.

To test the hypothesis that aberrant SoA in schizophrenia is induced by a TD in predictive signal transduction, we added random TDs to the information processing of the context units in the RNN model during the SoA task (see Eq. (6) in Methods). The TD in context unit lesions induced two different types of changes in SoA judgment. Figure 5a, b illustrates two representative examples of yes-rate plots. Figure 5a shows that yes-rates increased with a jumping latency of 400 to 1000 ms in the action-linked trial and with all conditions in the ‘event prior to action (EPA)’ trial but did not change with a jumping latency of 0 to 100 ms in the action-linked trial, illustrating the excessive SoA associated with PS. Figure 5b shows that yes-rates decreased with a jumping latency from 0 to 500 ms in the action-linked trial but did not change with a jumping latency from 800 to 1000 ms in the action-linked trial, illustrating the diminished SoA associated with NS.

### SL-ex2 (random noise)

We conducted three additional lesion experiments as the control experiments for our proposed hypothesis. In SL-ex2 (random noise), we modified the context unit activities. Random noise was added to the membrane potentials of all context units during the SoA task (see Eq. (7) in Methods). Figure 5c shows a representative example of yes-rate plots induced by SL-ex2 (random noise). ‘Yes’ rates during the short jumping latency conditions decreased, while ‘yes’ rates during the long jumping latency condition increased, indicating that SL-ex2 (random noise) induced random SoA judgment. This change in SoA was observed in all M-subjects.

### SL-ex3 (TD in output)

To investigate whether the observed effect of TD was specific to signal transductions between context units, we simulated the lesion experiment by adding a TD for signal transductions from all context units to the output unit corresponding to SoA judgment (see Eq. (8) in Methods). Figure 5d, e illustrates representative examples, which showed no change related to SoA judgment in SL-ex3 (TD in output).

### SL-ex4 (TD in input)

TD was applied to all signal transductions from input to context units (see Eq. (9) in Methods). Figure 5f shows yes-rate plots for a simulated lesion experiment. This lesion experiment induced random SoA judgment: ‘yes’ rates decreased during short jumping latency conditions and increased during long jumping latency conditions.

### Comparison between patients with schizophrenia and lesioned M-subjects

Among the four lesion experiments, only SL-ex1 replicated the behavioral patterns of patients with schizophrenia patients. Within SL-ex1, 10 M-subjects from the same HC were randomly reproduced into PS and NS types. Therefore, rather than independently verifying if each lesioned M-subject replicated schizophrenic behavioral data, we quantitatively evaluated the similarity between 50 schizophrenic behavioral data and the distribution of each lesion experiment.

**Fig. 6:**
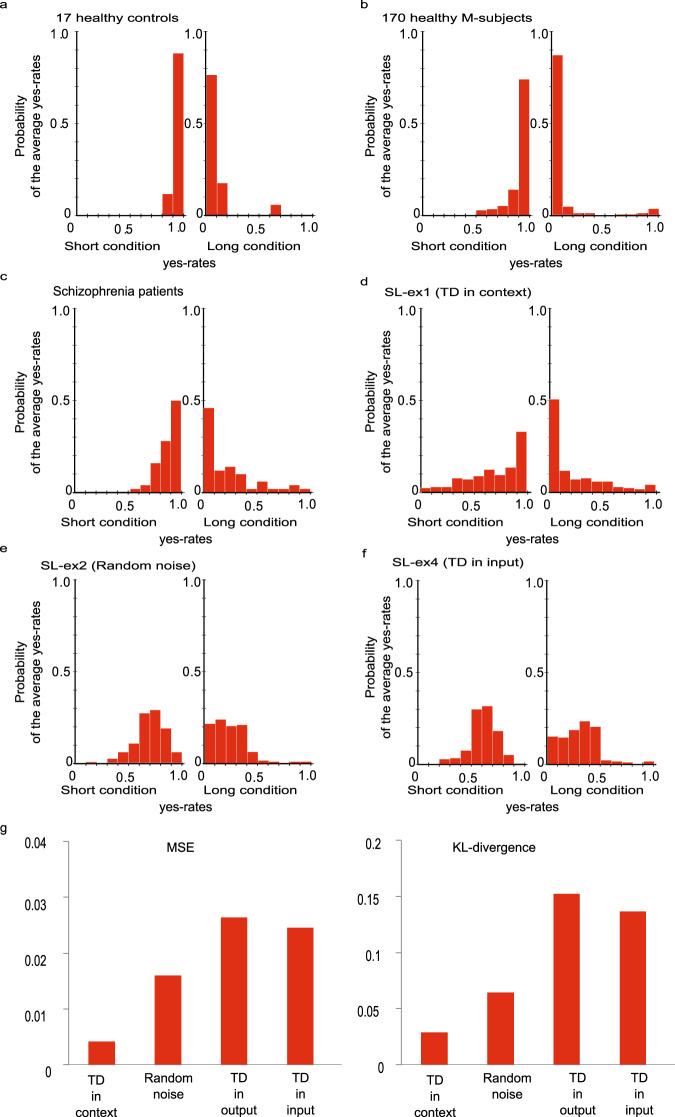
Similarity between behavioral data in patients with schizophrenia and performance of the lesioned M-subjects. **a** The distribution of the average ‘yes’ rate in the healthy control group (17 H-subjects). The horizontal axis shows the ‘yes’ rate within the short jumping latency condition (0 to 200 msec, left panel) and in the long jump latency condition (800 to 1000 msec, right panel). The vertical axis shows the probability of the average ‘yes’ rates. **b** The distribution of the average ‘yes’ rate in models for the healthy control group (170 M-subjects). **c** The distribution among patients with schizophrenia. **d** The distribution in SL-ex1 (TD in context). **e** The distribution in SL-ex2 (random noise). **f** The distribution in SL-ex4 (TD in input). **g** Mean square error (MSE) and Kullback–Leibler-divergence (KL-divergence), comparing the distributions of the behavioral data and that of each of the lesion experiments.

Maeda et al. ^7^ showed that the differences in behavioral data between HCs and patients with schizophrenia were particularly prominent in short (0–200 ms) and long jumping (800–1000 ms) latency conditions in action-linked trials. Therefore, we quantitatively analyzed the yes-rate values within these two intervals to reflect the behavioral characteristics of the subjects in the SoA task. Figure 6a, b shows distributions of the average yes-rates during short and long jumping latency conditions in 17 H-subjects and 170 M-subjects. In the short jumping latency condition, the average yes-rate probabilities had a high and leptokurtic peak at 1.0 on the horizontal axis, indicating that both H-subjects and M-subjects answered “yes” with high frequency. In the long jumping latency condition, the average yes-rate probabilities had a high and leptokurtic peak at 0.0 on the horizontal axis, indicating that both H-subjects and M-subjects answered “no” with high frequency.

The distribution of average yes-rates during short and long jumping latency conditions in schizophrenia patients is shown in Fig. 6c. For short jumping latency, the peak was 1.0 on the horizontal axis, similar to H-subjects. However, the probability of 1.0 was lower and the distribution was spread to approximately 0.5. The above feature resulted from the combination of the two types of schizophrenia: PS subjects answered “yes” with high frequency and NS subjects answered “yes” with a lower frequency (diminished SoA). Similarly, in the long jumping latency condition, the peak was 0.0 on the horizontal axis (similar to H-subjects). However, compared to H-subjects, the probability at 0.0 on the horizontal axis was lower, and the distribution was spread to around 0.4. This feature of the distribution also resulted from the combination of the two types of schizophrenia; that is, PS subjects answered “yes” with a higher frequency (excessive SoA), and NS subjects answered “no” with a frequency similar to H-subjects.

We compared the above features in patients with schizophrenia to the features of the four lesion experiments. As a qualitative comparison, we illustrated the distributions of the average ‘yes’ rates in simulated lesion experiments (Fig. 6d–f; the distribution in SL-ex3 [TD in output] was not illustrated because there was no change in SoA judgment in healthy M-subjects). The distribution in SL-ex1 (TD in context) seemed to be most similar to that observed in patients with schizophrenia. Meanwhile, in the distributions of SL-ex2 (random noise) and SL-ex4 (TD in input), the peak was at around 0.5 on the horizontal axis, resulting from random SoA judgments induced by the lesion experiments.

**Fig. 7:**
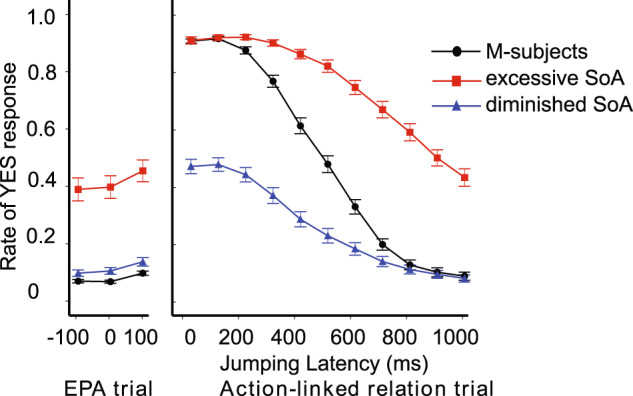
Schizophrenia-pattern aberrant SoA in SL-ex1 (TD in context). The temporal delay in context units (SL-ex1 [TD in context]) induced two different types of changes with regard to SoA judgment, similar to behavioral data in patients with schizophrenia (Fig. 1c). Bars represent the standard error.

To quantitatively evaluate the similarity between the distributions of the behavioral data and that of each lesion experiment, we calculated the mean squared error (MSE) and the Kullback–Leibler-divergence (KL-divergence)^30^, which are distance measures of probabilistic distributions. As shown in Fig. 6g, SL-ex1 (TD in context) had the lowest values in both indices among the four lesion experiments, and hence was the best fit for the behavioral data. SL-ex1 (TD in context) induced two different types of changes in SoA judgment among patients with schizophrenia. As such, we attempted to quantitatively classify the schizophrenia-pattern changed M-subjects in SL-ex1 (TD in context) into two groups (see Methods as for detailed classifying procedures). The ‘yes’ rates in the two groups were similar to those of the behavioral data (Figs. 1C, 7). However, there are dissimilarities between Fig. 1c and Fig. 7. Notably, there is a pronounced decline in the yes-rate under the short-delay conditions in the NS group in Fig.7. Statistical test revealed significant differences between yes rates in Fig. 1c and in Fig. 7 (see Supplementary Table 4).

## Discussion

In this study, we proposed a computational model of SoA judgment as a predictive processing system using an RNN model. The proposed model successfully reproduced the behavioral features of HCs in the SoA task. In addition, a schizophrenia-pattern aberrant SoA was reproduced in the lesion experiment by adding a TD in signal transfer to the predictive processing network. Schizophrenia-pattern aberrant SoA was not observed in the three other lesion experiments. These results support the hypothesis that a TD in predictive signal transduction causes malfunctioning SoA in schizophrenia patients.

Understanding how the proposed RNN model works may provide insights into understanding the mechanisms of the brain network for SoA judgment and its vulnerability to schizophrenia. The first question is how the RNN model judges the SoA and maintains the time of jumping latency. To address this question, correlations between SoA judgment, jumping latency condition, and context unit activities immediately before the piece jumps in terms of the predictive processing system were evaluated. The mean number of context units that significantly correlated with SoA judgment was 9.497/10 units (Mann–Whitney *U* test, *p* < 0.05), and the mean number of context units that significantly correlated with the jumping latency condition was 9.512/10 units (Spearman’s rank-order correlation coefficient, *p* < 0.01). This analysis indicated that almost all context units were dispersedly involved in SoA judgment and the maintenance of the time of jumping latency. These observations are consistent with recent studies demonstrating that the functions of SoA, including the predictive component and comparator, are not located in a specific brain region, but may be represented as functional connectivity of several brain regions as an agency network^12,31^.

The second question relates to which features of the RNN model are vulnerable to schizophrenia-pattern changes. Neither the vulnerability nor the excessive/diminished SoA were related to the behavioral characteristics of the H-subject (i.e., training data) because 10 M-subjects trained against the same H-subject randomly showed vulnerability to schizophrenia and both types of SoA changes (see Supplementary Table 1). In addition, we attempted to analyze the characteristics of network structures. When we compared the connective weights of the schizophrenia-pattern change and the no-change groups as an assessment of the structural features in our RNN model, we could not find any significant differences in vulnerability to schizophrenia (see Supplementary Table 2). Therefore, we focused on analyzing the dynamics of the RNN model associated with schizophrenia-pattern changes in SoA in SL-ex1 (TD in context). We calculated the mean number of context units functionally involved in SoA judgment (see Supplementary Table 3). At the stage around the timing of the jump, the number of SoA-related context units in the lesioned M-subjects was significantly less than in healthy M-subjects, implying that the TD lesion in the predictive processing system was related to the dispersible function decrease. This result provides valuable insight into the abnormalities of functional connectivity (disconnection hypothesis of schizophrenia) in the brains of patients with schizophrenia^24,32–35^, as we found that the TD in the predictive processing system itself, rather than in the input or output processes, may be responsible for functional disconnection in schizophrenia.

In the current model, the temporal delays would cause reduction in the accumulation of activities of context units, resulting in schizophrenia-pattern aberrant changes in SoA. To provide collateral evidence of this explanation, we conducted an additional lesion experiment in which we reduced the value of the gain parameter (see Additional lesion experiment (reduced gain) in Methods). The reduction could be expected to induce a decrease in the accumulation of the activities of context units, that is, a decrease in the change in the activities of context units. The results showed that the MSE was 0.0059 and KL-divergence was 0.0300 between the distribution of the behavioral data and the additional lesion experiment (reduced gain), which were similar to the MSE (0.0042) and KL-divergence (0.0287) in SL-ex1 (TD in context; Supplementary Fig. 2a, b). Therefore, the results suggested that reducing the value of the gain parameter induced a schizophrenia-pattern change in SoA (Supplementary Fig. 2c, d).

This is consistent with a previous theoretical study claiming that the reduced gain parameter value is associated with the decreased dopaminergic function and the dysfunction of the prefrontal cortex in schizophrenia^36^. In addition, some biological studies suggest that impaired white matter may be pathophysiologically related to dysfunction of the glutamate system and aberrant SoA in schizophrenia^37–39^. Overall, in a computational sense, TD can be considered as another route or a parallel phenomenon of functional disconnection in schizophrenia. Our computational approach study gives a neuro-dynamical explanation, linking the TD in the predictive processing system to the abnormal myelination in the frontoparietal network observed by Whitford^17–20^, with the functional disconnection hypothesis and the abnormality of gain parameter affecting the degree of activities of context units and represented with the impairment of dopamine and cognitive function^36^ in the mechanism of aberrant SoA in schizophrenia. Moreover, brain oscillations are considered vital biomarkers for understanding the integration of top-down predictions and bottom-up input^40,41^. Context units in the RNN model synchronize current external inputs with past information. SL-ex1 may disrupt the oscillations, linking to altered oscillations and the hypothesized top-down delay.

Although we suggest a mechanism of schizophrenia-pattern aberrant changes, why the contradictory phenomenon (i.e., the excessive/diminished SoA) occurs is still unclear. This is a limitation of our study. However, our result of a single lesion in the network inducing a contradictory phenomenon suggests potential impacts on the pathophysiology, diagnostics, therapies, and prognostics of schizophrenia. Sophisticated analytical tools and methodologies could deepen our comprehension.

This constitutes one of the most controversial problems in SoA studies of schizophrenia^7,42^. We have explained the contradictory phenomenon from the standpoint of compensatory contribution of retrospective components in SoA judgment. Under abnormal predictive components, patients with paranoid-type schizophrenia may rely on retrospective components including inference based on contextual information. However, patients with negative symptom-predominant schizophrenia may not utilize those retrospective components, resulting in a state in which they can hardly feel causal efficacy to the external environment^6,7^.

The current study has several limitations. First, although we attempted to conduct a yes-rate plot as a means of comparing actual similarities between the behavioral data from 50 schizophrenia patients and SL-ex1, the statistical test revealed significant differences between yes rates in Fig. 1c and in Fig. 7. The model can only apply temporal delays in 1-timestep increments, which makes it difficult to finely adjust the magnitude of the effect temporal delays. This may contribute to the discrepancies between the model and human data. Moreover, in actual human judgment, there may be a strong bias towards responding ‘yes’ in short latency conditions, even in the presence of signal anomalies. This suggests a tendency to maintain ‘yes’ responses for short latency. However, the current model does not account for such bias. Consequently, SL-ex1 is more likely to exhibit transitions from “yes” to “no” during short latency conditions than actual schizophrenia patients (Fig. 6c, d). Nonetheless, we consider that these statistical differences do not detract from the significance of our study. The most crucial observation in this study is that SL-ex1 qualitatively reproduces the schizophrenia-pattern of SoA judgement, characterized by excessive/diminished SoA. We have indeed confirmed that the SL-ex1 successfully replicated the bi-directional behavioral changes at the individual M-subjects level (Fig. 5a, b), as well as in the MSE and KL-divergence analysis and scatter plots in Supplementary Fig. 3. Second, Yano et al. ^43^ emphasized that the prediction of the time of jumping latency in the Keio method should be considered as a learning process. However, in our study, the model was trained with a ‘batch’ learning schema, and the effect of learning during the experiments was ignored. Similarly, Legaspi and Toyoizumi^44^ proposed a computational model of SoA using a Bayesian inference approach, and emphasized the effect of uncertainty estimation of sensory stimuli in SoA judgements. Again, the current neural network model did not implement uncertainty estimation of sensory inputs. Integration with knowledge from the Bayesian aspects^45^ may provide further insights for the biological study of the pathological mechanism of aberrant SoA in schizophrenia. Indeed, a stochastic continuous time RNN model^46^, equipping uncertainty in sensory inputs and predictive coding-inspired variational RNN^47^, and equipping uncertainty in hidden states in the free energy principle^48^, have been developed and applied to computational psychiatry^49–51^. Another technical limitation of the present study is that we heuristically determined the number of context units. A larger number of context units in the algorithm increases network learning precision, but at the cost of a tendency for the network to overlearn^52^. We computed the training and generalization errors using the root mean square error between the yes rate of SoA in the healthy control behavioral data and the reproduction and test phase of the RNN model, respectively. There was no significant difference between the two errors (Mann–Whitney *U* test, *p* = 0.748), meaning the RNN model did not overlearn. In addition, while free parameters (such as a and τ) were meticulously optimized through an exploratory process to reproduce the behavioral data of HCs, these values lack a foundation of biological plausibility.

In conclusion, we examined the hypothesis that a TD in predictive signal transduction causes malfunctioning SoA through a computational modeling experiment. Our results demonstrated that a TD in signal transduction in the RNN model resulted in aberrant SoA through an impaired information integration process. The present study provides a mechanistic understanding of the pathology of altered SoA in patients with schizophrenia. Our methodology of network modeling opens the door to further studies of aberrant SoA, including brain imaging and physiological studies.

## Methods

### Behavioral data for SoA

The explicit SoA task (Keio method) evaluates the experience of the temporal causal relation between an intentional action and an external event (Fig. 1a, b). Figure 1a shows an overview of the task referred to as an ‘action-linked relation.’ In each trial, a square piece appeared from the bottom of the screen and moved upward at a uniform speed. Subjects were instructed to press a button with their finger immediately after they heard a specific sound. When the subject pressed the button, the piece jumped upward with latencies ranging from 0–1000 ms, in 100 ms increments. The jumping latency was randomly shuffled for each trial. After each trial, the subjects judged their SoA with a “yes” or “no” response. Each jumping latency condition was run 10 times. In addition to the action-linked trials, ‘event prior to action (EPA)’ trials were conducted, in which the piece jumped in connection with the sound (Fig. 1b); EPA trials were performed under three conditions (100 ms before the beep, simultaneously with the beep, or 100 ms after the beep). Each EPA trial was also repeated 10 times. Each experiment included a random mixture of action-linked and EPA trials. There were a total of 140 trials per subject.

Patients with schizophrenia showed bidirectional changes in SoA compared to HCs in a previous study^7^. Statistically significant differences were demonstrated using an ANOVA (see Supplementary Table 5 for more details of the statistical analysis. Figure 1c depicts the results from Maeda et al.‘s study^7^).

### Recurrent neural networks

To investigate the pathological mechanisms of altered SoA in schizophrenia, we developed a macro-level neural dynamics model as a dynamical system of information processing for the SoA task. The main element of the model is a continuous-time RNN^24,53^, which is often used for modeling temporal sequence learning^54–56^. The sensory inputs to the system were visual (object position), auditory (beep sound), and proprioceptive (button press). The outputs were sensory input predictions and judgment of SoA. An example of a trial sequence used in the simulation is shown in Fig. 3. Additionally, there were ten context units with recurrent inputs to every context unit (including itself). A schematic drawing of the temporal dynamics with the context units of the RNN is shown in Supplementary Fig. 1. Input and output units were connected only via context units.

The neuronal model is a conventional firing rate model in which each unit’s activity represents the average firing rate over a group of neurons. The continuous time characteristics of the RNN model were described by differential equations and computed according to numerical approximations, as follows:

1 $${u}_{i}\left(t\right)=\left(1-\frac{1}{\tau }\right){u}_{i}\left(t-1\right)+\frac{1}{\tau }\left[\sum _{j\in {Input}}{w}_{{c}_{i}{x}_{j}}{x}_{j}\left(t\right)+\sum _{k\in {Context}}{w}_{{c}_{i}{c}_{k}}{c}_{k}\left(t-1\right)\right]$$

2 $${c}_{i}\left(t\right)=f\left({u}_{i}\left(t\right)\right),i\in {Context}\,(i=1\ldots 10)$$

where ${u}_{i}\left(t\right)$ is the membrane potential of the *i*th context unit for the current time step $t$; ${x}_{j}\left(t\right)$ is the state of the *j*th input unit (corresponding to the object position, beep, or button press); and ${w}_{{AB}}$ is the synaptic weight from *unit B* to *unit A*. The first term in Eq. (1) corresponds to the history of the internal state, the second term corresponds to the effects of current external inputs (${x}_{j}\left(t\right)$) and recurrent inputs (${c}_{k}\left(t-1\right)$) through the context units, respectively. This means that the membrane potential of the context unit is assumed to be influenced by current inputs and their previous state, enabling the RNN model to reproduce complex task sequences and predict future states. The time constant $\tau$ is defined as the decay rate of the membrane potential of the unit; $\tau$ was set to 10.0. ${c}_{k}\left(t\right)$ is the state of the *k*th context unit and is calculated using the following activation function:

3 $$f\left(u\right)=\frac{1}{1+\exp (-{au})}$$

Equation (3) is a conventional sigmoid function, and $a$ is a gain parameter. In this study, $a$ was set to 1.0.

The temporal dynamics of output units are shown in the following equations,

4 $${u}_{i}\left(t\right)=\sum _{j\in {Context}}{w}_{{o}_{i}{c}_{j}}{c}_{j}\left(t-1\right),i\in {Output}$$

where the membrane potential of the *i*th output unit, ${u}_{i}\left(t\right)$ (corresponding to object position, beep, button press, or judgment of SoA), accepts signals only from context units at the previous time step, $t-1$. The state of the *i*th output unit, ${o}_{i}\left(t\right)$, is computed with the following equation:

5 $${o}_{i}\left(t\right)=f\left({u}_{i}\left(t\right)\right),i\in {Output}$$

Free parameters (such as a and τ) were meticulously optimized through an exploratory process to reproduce the behavioral data of HCs.

### Experimental procedure: training of the RNN model and lesion experiments

The experimental procedure is illustrated in Fig. 2. The behavioral data utilized in this study were not newly acquired; they were derived from a subset of the behavioral experiment data in a previous study^7^, conducted and recruited at Keio University and Sakuragaoka Memorial Hospital in Japan. The subjects’ characteristics for each group in the current study are as follows: HCs consist of 17 individuals (males: 11, females: 6; mean age: 33.6 years, S.D. = 7.1), selected from the 35 subjects of the study by Maeda et al. ^7^, whose data of the response reaction time are available (which is essential information for the simulations in our current study). Patients with Schizophrenia encompassed the complete dataset of 50 individuals from the study by Maeda et al. ^7^. The diagnosis of schizophrenia was based on the DSM-IV diagnostic criteria. Among the patients diagnosed with schizophrenia, those with prominent negative symptoms were designated as NS (20 individuals; males: 12, females: 8; mean age: 36.2 years, S.D. = 10.0) on the Positive and Negative Syndrome Scale (PANSS);^57^ the rest were denoted as PS (30 individuals; males: 21, females: 9; mean age: 37.9 years, S.D. = 11.9). We conducted statistical analysis of the data to ensure whether the age and gender of the subjects matched across the groups. The Chi-squared test for gender distribution and the ANOVA for age distribution showed no statistically significant differences among the three groups. We trained RNN models against the actual behavioral data of 17 HCs (referred to as H-subjects). Trained RNN models were treated as simulated subjects in the experiments (referred to as M-subjects). A total of 140 sequences corresponding to 140 behavioral trials per H-subject were used for training RNN models. We generated 10 M-subjects from each H-subject; generating 170 M-subjects in total. A conventional backpropagation through time (BPTT) algorithm was implemented for training the RNN model^53,58^. After training the M-subjects, we checked whether each M-subject successfully reproduced the behavioral features of the SoA task in the corresponding H-subject. We *newly* created the test sequences for the reproduction test as follows: 100 trial sequences for each condition of jumping latency were created, resulting in 1400 trial sequences (randomly shuffled) for the reproduction test in a single M-subject. Reaction times (RTs) of button press for the test sequences were sampled based on the assumption of a normal distribution whose mean and standard deviation (SD) were set to the average and SD of the RTs of the corresponding H-subjects, respectively.

To examine the hypothesis that temporal delays in predictive signal transduction cause aberrant SoA in schizophrenia, we conducted a simulated lesion experiment with M-subjects (SL-ex1 [TD in context]). In this experiment, we modified the membrane potential of the context unit in Eq. (1), as follows:

6 $${u}_{i}\left(t\right)=\left(1-\frac{1}{\tau }\right){u}_{i}\left(t-1\right)+\frac{1}{\tau }\left[\sum _{j\in {Input}}{w}_{{c}_{i}{x}_{j}}{x}_{j}\left(t\right)+\sum _{k\in {Context}}{w}_{{c}_{i}{c}_{k}}{c}_{k}\left(t-1-\Delta \right)\right]$$

$$i\in {Context}\,(i=1\ldots 10)$$

${c}_{k}\left(t-1\right)$ in Eq. (1) was replaced with ${c}_{k}\left(t-1-\Delta \right)$, where Δ was assumed to correspond to the temporal delay of predictive signal transduction. The value of Δ for each context unit was randomly changed between 0 and 1 over time. This was done to eradicate any potential arbitrariness concerning the moment of introducing the temporal delay into individual context units and juxtaposing this methodology with the three lesion experiments detailed subsequently, serving as controls.We conducted three further lesion experiments as controls. The overview of each experiment is as follows. In Eq. (1), ${c}_{k}\left(t-1\right)$ signifies RNNs’ recurrent inputs, incorporating past sensory input history. SL-ex1 explores the TD hypothesis by directly introducing TD signals to predictive signal transduction. SL-ex2 simulates simple disruption of historical sensory input information by adding random noise to the internal states of the context unit. SL-ex3 depicts accurate processing of historical sensory input information but impaired output reflection; SL-ex4 portrays a situation where only current sensory inputs experience TD.

In the second lesion experiment (SL-ex2 [Random noise]), random noise was added to the internal states of the context units instead of temporal delay:

7 $${u}_{i}\left(t\right)=\left\{\left(1-\frac{1}{\tau }\right){u}_{i}\left(t-1\right)+\frac{1}{\tau }\left[\sum _{j\in {Input}}{w}_{{c}_{i}{x}_{j}}{x}_{j}\left(t\right)+\sum _{k\in {Context}}{w}_{{c}_{i}{c}_{k}}{c}_{k}\left(t-1\right)\right]\right\}\times \left(1+{additive}\,{noise}\right)$$

$$i\in {Context}\,(i=1\ldots 10)$$

the internal state value of each neuron ${u}_{i}\left(t\right)$ was modified into ${u}_{i}\left(t\right)\times (1+{additive}\,{noise})$ in Eq. (1). The level of additive noise was set to 0.01.

In the third lesion experiment (SL-ex3 [TD in output]), temporal delay was selectively applied for the signals between context units and the SoA output unit:

8 $${u}_{{SoA}{unit}}\left(t\right)=\sum _{j\in {Context}}{w}_{{SoAunit}{c}_{j}}{c}_{j}\left(t-1-\Delta \right)$$

${c}_{j}\left(t-1\right)$ in Eq. (4) was replaced with ${c}_{j}\left(t-1-\Delta \right)$. Δ was randomly changed from 0 to 1 over time.

As the fourth lesion experiment (SL-ex4 [TD in input]), a temporal delay was selectively applied for the signals between input units and context units:

9 $${u}_{i}\left(t\right)=\left(1-\frac{1}{\tau }\right){u}_{i}\left(t-1\right)+\frac{1}{\tau }\left[\sum _{j\in {Input}}{w}_{{c}_{i}{x}_{j}}{x}_{j}\left(t-\Delta \right)+\sum _{k\in {Context}}{w}_{{c}_{i}{c}_{k}}{c}_{k}\left(t-1\right)\right]$$

$$i\in {Context}$$

${x}_{j}\left(t\right)$ in Eq. (1) was replaced by ${x}_{j}\left(t-\Delta \right)$. Δ was randomly changed from 0 to 1 over time.

To compare actual behavioral data^6,7^ in patients with schizophrenia and that in M-subjects simulated by the four experiments, we examined the mean squared error and the Kullback–Leibler-divergence, which are distance measures of probabilistic distributions.

This study was approved by the Ethics Committee of Sakuragaoka Memorial Hospital on August 15, 2018, and Keio University (No: 2010-0202-6). The study was conducted in accordance with all the relevant guidelines and regulations.

### Quantitative classification of the lesioned M-subjects in SL-ex1 (TD in context) into two groups

We quantitatively classified the 170 lesioned M-subjects in SL-ex1 (TD in context) through ${Y}_{i}$:

10 $${Y}_{i}=\sum _{j}\left({{x}_{l}}_{i,j}-{{x}_{h}}_{i,j}\right)/3$$

where ${{x}_{l}}_{i,j}$ is the ‘yes’ rate in jumping latency time *j* in lesioned M-subject No. *i*, *j* = 0, 100, 200 within the short jumping latency condition and *j* = 800, 900, 1000 within the long jumping latency condition, and ${{x}_{h}}_{i,j}$ is the yes-rate during jumping latency time *j* in a test of *i*-th healthy M-subject. We show scatter plots for ${Y}_{i}$ in SL-ex1 (TD in context) and SL-ex2 (random noise) in Supplementary Fig. 3a, b, respectively. The distribution in SL-ex1 (TD in context) showed expansions in two directions, in contrast to SL-ex2 (random noise), meaning that SL-ex1 (TD in context) induced two different types of changes in SoA judgment. We classified the data in SL-ex1 (TD in context) with the -2 thresholds in the short jumping latency condition and the 2 with the long jumping latency condition; we defined the upper-right part of the distribution as excessive SoA and the lower-left part as diminished SoA. We referred to M-subjects with both excessive and diminished SoA as the schizophrenia-pattern change group, and to the residual M-subjects as the no-change group.

### Additional lesion experiment (reduced gain)

In Eq. (1), the term, $\sum _{k\in {Context}}{w}_{{c}_{i}{c}_{k}}{c}_{k}\left(t-1\right)$, is the information from the past activities of context units to the current membrane potential of the context unit. In SL-ex1 (TD in context), we modified Eq. (1) of the membrane potential of the context unit as Eq. (6). Because the information from the past activities of context units is delayed ($\sum _{k\in {Context}}{w}_{{c}_{i}{c}_{k}}{c}_{k}\left(t-1-\Delta \right)$) in Eq. (6), the change of the membrane potential of the context unit (${u}_{i}\left(t\right)$) deaccelerates. For example, if $\sum _{k\in {Context}}{w}_{{c}_{i}{c}_{k}}{c}_{k}\left(t-1\right)$ in Eq. (1) is increasing, $\sum _{k\in {Context}}{w}_{{c}_{i}{c}_{k}}{c}_{k}\left(t-1-\Delta \right)$ in Eq. (6) is less than $\sum _{k\in {Context}}{w}_{{c}_{i}{c}_{k}}{c}_{k}\left(t-1\right)$ in Eq. (1). Therefore, a building-up of the change of ${u}_{i}\left(t\right)$ decreases. These decreases in accumulation of activities of context units (i.e., build-ups or damping) could be related to schizophrenia-pattern aberrant changes in SoA.

To provide collateral evidence of this explanation, we made an additional lesion experiment reducing the value of gain parameter in Eq. (3). The gain parameter, denoted as “a”, is a free parameter. By assigning a value of a = 1, we successfully reproduced the healthy behavior of the subjects. In the lesion experiments, the value of “a” remained unchanged from that of the healthy model to analysis the only influence of TD. In the additional lesion experiment (reduced gain), we exclusively altered the numerical value of “a”. Equation (3) is a conventional sigmoid function, and a gain parameter, $a$, affects the degree of activities of context units by changing a slope of the sigmoid function. Reducing the value of the gain parameter makes the slope of a sigmoid function shallower and can be expected to induce a decrease in the accumulation of the activities of context units, that is, the decrease in the change in the activities of context units. Three patterns were tested for “a”: 0.99, 0.9, and 0.5 in Eq. (3). Among these patterns, the value of 0.99 fits the behavioral data of Schizophrenia.

### Supplementary information

Supplementary Information

## Data Availability

The data that support the findings of this study and the analysis code are available from the corresponding author upon reasonable request.

## Code availability

The training codes are available from the corresponding author upon reasonable request.
