# Supplementary material for: Aberrant sense of agency induced by delayed prediction signals in schizophrenia: a computational modeling study
Source: Schizophrenia (Heidelb). 2023 Oct 16;9(1):72. doi: 10.1038/s41537-023-00403-7 (PMC10579420; doi:10.1038/s41537-023-00403-7)
Supplement: Supplementary file 1 — Supplementary Information [file 41537_2023_403_MOESM1_ESM.docx]

**SUPPLEMENTARY INFORMATION**

**for**

**Aberrant sense of agency induced by delayed prediction signals in schizophrenia: a computational modeling study**

**SUPPLEMENTARY FIGURES**


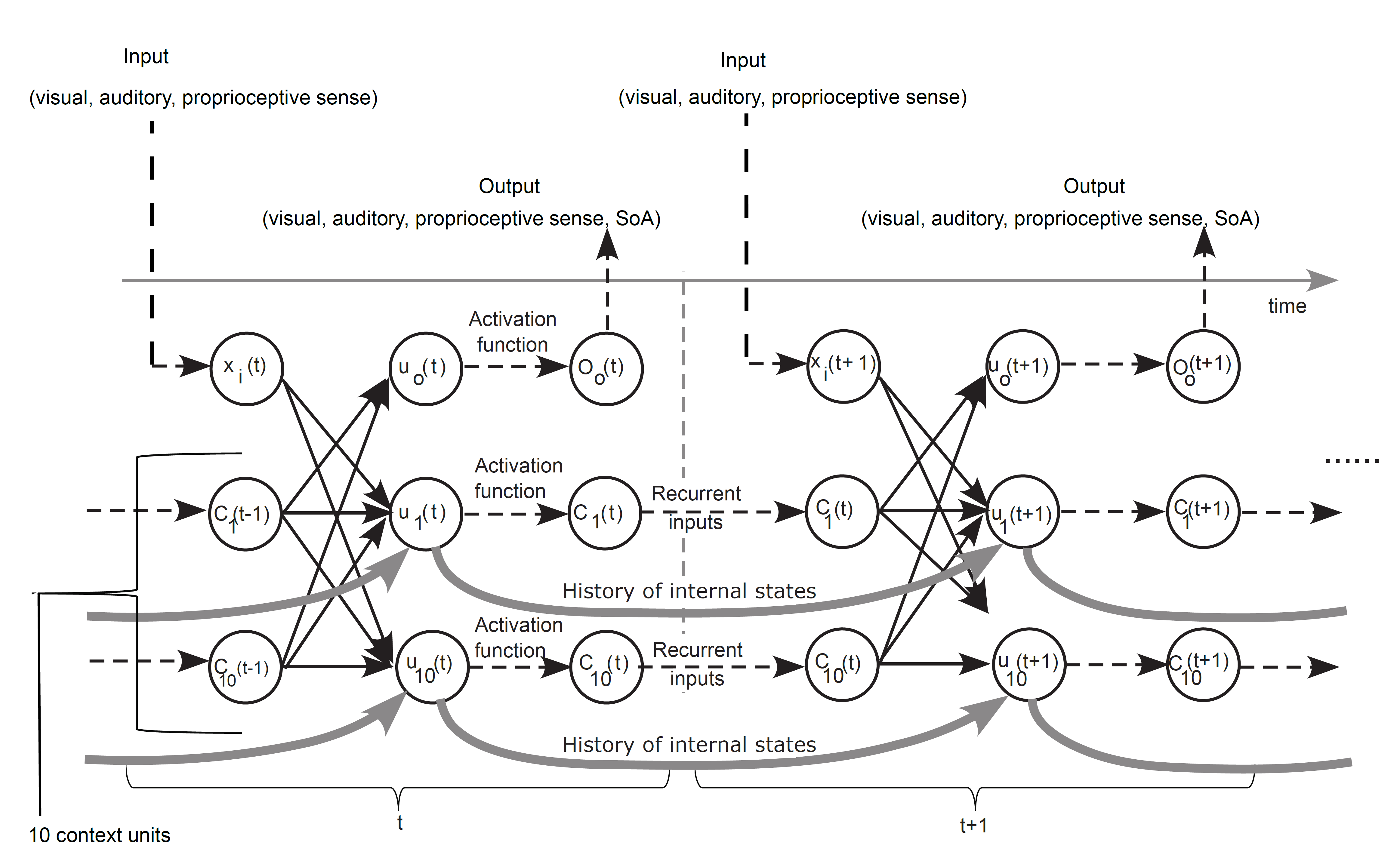


**Supplementary Fig. 1 Overview of the recurring neural network (RNN) model.** The RNN model is composed of three input units: visual sense, auditory sense, and proprioceptive sense (notating each state as $x_{i}\left( t \right)$); four output units: predictions of visual sense, auditory sense, proprioceptive sense, and judgment of SoA (notating each state as $o_{o}\left( t \right)$); ten context units (notating each state as $c_{k}\left( t \right)$). $u_{k}\left( t \right)$ is the membrane potential of the context unit, $u_{o}\left( t \right)$ is the membrane potential of the output unit, and $t$ is the current time step. Based on the history of the internal state (i.e., the previous $u_{k}\left( t-1 \right)$) and synaptic inputs (i.e. the current $x_{i}\left( t \right)$ and the previous $c_{k}\left( t-1 \right)$), the model generates the current $u_{k}\left( t \right)$ for the context unit. Then $c_{k}\left( t \right)$ is calculated through the activation function and copied as the recurrent input and used to generate the future $u_{k}\left( t+1 \right)$. The membrane potential $u_{o}\left( t \right)$ of the output unit $o_{o}\left( t \right)$ is generated based only on the previous $c_{k}\left( t-1 \right)$, and $o_{o}\left( t \right)$ is calculated through the activation function.

**
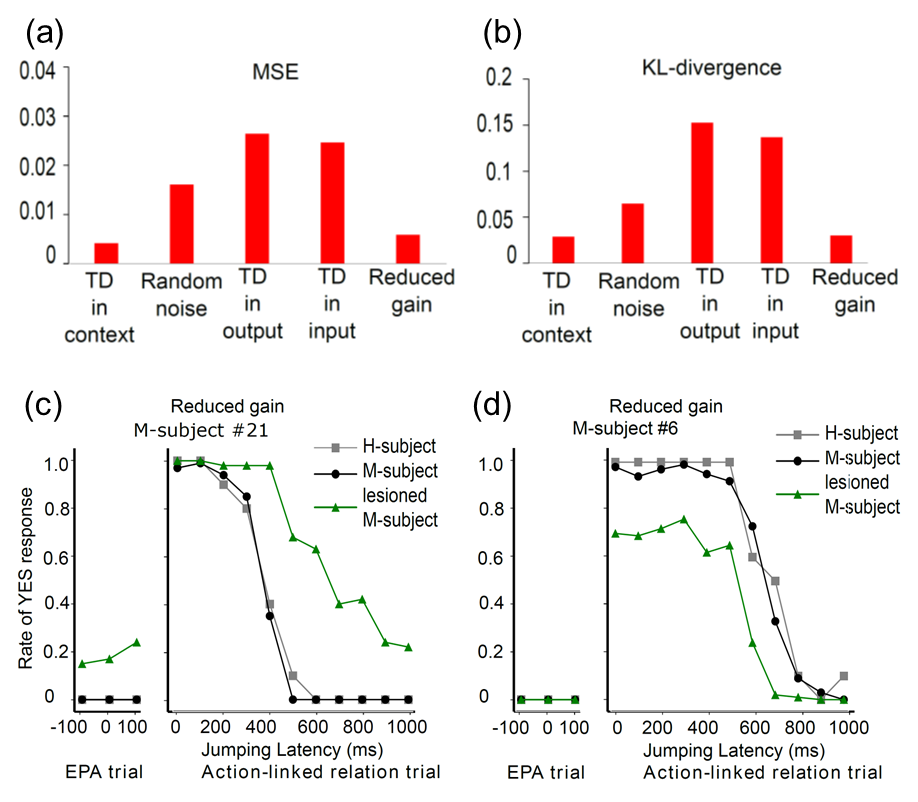
**

**Supplementary Fig. 2 Additional lesion experiment (reduced gain).** (**a & b**) Similarity between behavioral data in patients with schizophrenia, and performance in the lesioned model subjects (M-subjects) including the additional lesion experiment (reduced gain). (**c**) An example of the excessive-type change in SoA judgment. (**d**) An example of the diminished-type change in SoA judgment.


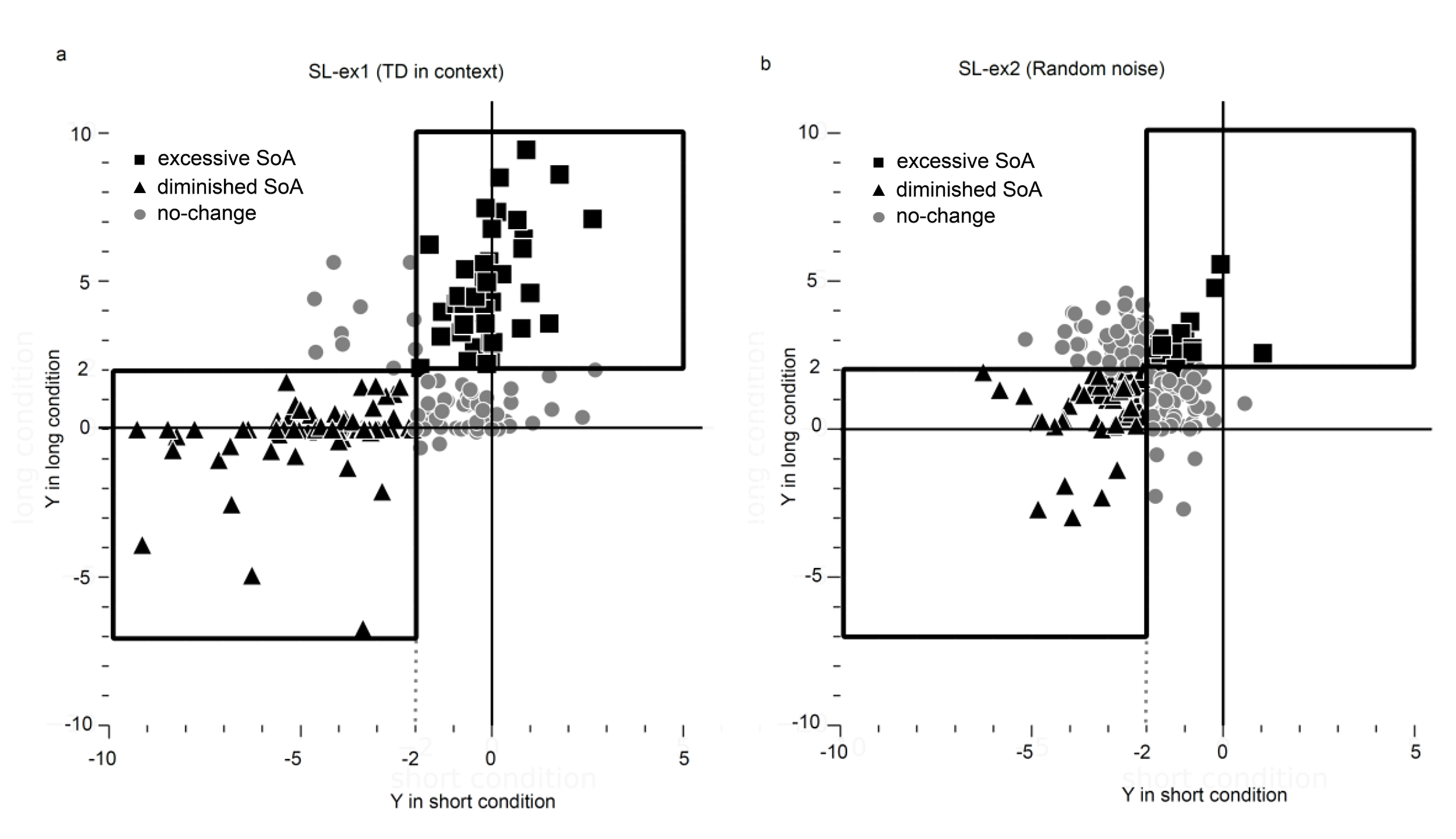


**Supplementary Fig. 3 The temporal delay in context units induced two different types of changes with regard to sense of agency (SoA) judgment, similar to behavioral data in patients with schizophrenia.** (**a & b)** Scatter plots (SL-ex1 [TD in context] and SL-ex2 [random noise], respectively), with $Y$ defined as $Y_{i}$ in equation (10). The horizontal axis shows $Y_{i}$ in the short jumping latency condition (written as short condition) and the vertical axis shows $Y_{i}$ in the long jumping latency condition (written as long condition).

**SUPPLEMENTARY TABLES**

**Supplementary Table 1. Associations between behavioral data of healthy controls (HCs) and schizophrenia-pattern changes in SL-ex1 (TD in context).** The simulated SL-ex1 (TD in context) randomly induced schizophrenia-pattern changes in sense of agency (SoA) for 10 model subject (M-subjects), which were generated based on the behavioral data of the same healthy subject (H-subject). If vulnerability to schizophrenia and excessive/diminished SoA resulted from the behavioral characteristics of the H-subject, the 10 M-subjects based on the same H-subject must change consistently by the SL-ex1 (TD in context). Therefore, the results in Supplementary Table 1 suggests that the schizophrenia-pattern change in SoA induced by SL-ex1 was not determined by the behavioral features of the H-subject.

| **HCs** | **Changes in SL-ex1 (TD in context)** | | |
| --- | --- | --- | --- |
| **No.** | **Excessive SoA** | **Diminished SoA** | **No change** |
| 1 | 2 | 5 | 3 |
| 2 | 5 | 1 | 4 |
| 3 | 3 | 6 | 1 |
| 4 | 3 | 3 | 4 |
| 5 | 0 | 3 | 7 |
| 6 | 2 | 6 | 2 |
| 7 | 4 | 3 | 3 |
| 8 | 3 | 2 | 5 |
| 9 | 2 | 3 | 5 |
| 10 | 0 | 5 | 5 |
| 11 | 4 | 3 | 3 |
| 12 | 3 | 4 | 3 |
| 13 | 4 | 4 | 2 |
| 14 | 0 | 4 | 6 |
| 15 | 1 | 5 | 4 |
| 16 | 2 | 5 | 3 |
| 17 | 4 | 4 | 2 |

**Supplementary Table 2. Associations between connective weights in the recurrent neural network (RNN) model and schizophrenia-pattern changes.**

|  |  | **Sc** | **No Change** | **P value** |
| --- | --- | --- | --- | --- |
| **Cx to SoA** | **mean** | 0.023 | -0.002 | 0.5 |
| **Cx to SoA** | **abs mean** | 1.003 | 1.072 | 0.331 |
| **Cx to SoA** | **var** | 1.948 | 2.318 | 0.125 |
| **Cx to SoA** | **abs var** | 0.959 | 1.08 | 0.252 |
| **Cx to Cx** | **mean** | 0.16 | 0.129 | 0.135 |
| **Cx to Cx** | **abs mean** | 3.2 | 3.28 | 0.421 |
| **(Cx self)-(Cx not self)** | **mean** | 0.108 | 0.321 | 0.425 |
| **(Cx self)-(Cx not self)** | **abs mean** | 0.905 | 1.076 | 0.364 |
| **Cx to Cx** | **var** | 18.334 | 18.768 | 0.438 |
| **Cx to Cx** | **abs var** | 6.677 | 6.809 | 0.371 |

Cx to SoA, connective weights from context units to the SoA judgment unit; Cx to Cx, connective weights from context units to other context units; Cx self, the connective weight from one of the context units to the same context unit (e.g., from context unit 1 to context unit 1); Cx not self, connective weights from one of the context units to the other context units (e.g., from context unit 1 to context unit 2~10); mean, mean of connective weights; abs mean, mean of absolute values of connective weights; var, variance of connective weights; abs var, variance of absolute values of connective weights; Sc, the schizophrenia-pattern change group; no change, the no-change group. The Mann–Whitney U test was used to compare differences between the two groups.

|  | Number of Cx units | | P value |
| --- | --- | --- | --- |
|  | Healthy  M-subjects | Temporal delay  in Cx units |  |
| Just before jump time | 9.497 | 9.133 | *0.008 |
| Jump + 10 steps | 9.645 | 9.349 | *0.014 |
| Just before  SoA judgement | 9.799 | 9.7659 | 0.414 |

**Supplementary Table 3. Correlation of the mean number of context units with sense of agency (SoA).**

We calculated the number of context units (Cx units) whose activities were correlated significantly with SoA judgment in three time steps (just before jump time, 10 time steps after jump time, and just before SoA judgment) in healthy M-subjects and in SL-ex1 (TD in context). *P < 0.05.

**Supplementary Table 4. Comparison between patients with schizophrenia and lesioned M-subjects in SL-Ex1.**

| **ANOVA TABLE** | | | | |
| --- | --- | --- | --- | --- |
|  | **F** | **df** | **P value** | **partial η^2^** |
| **Subject** | 0.46 | (1, 353) | 0.50 | 0.001 |
| **Group** | 152.43 | (2, 353) | < .001* | 0.46 |
| **Temporal Delay** | 587.36 | (13, 4589) | < .001* | 0.62 |
| **Subject x Group** | 5.57 | (2, 353) | < .05* | 0.03 |
| **Subject x Temporal Delay** | 15.31 | (13, 4589) | < .001* | 0.04 |
| **Group x Temporal Delay** | 31.16 | (26, 4589) | < .001* | 0.15 |
| **Subject x Group x Temporal Delay** | 5.20 | (26, 4589) | < .001* | 0.03 |

| **Simple main effects of subject within each group** | | | | |
| --- | --- | --- | --- | --- |
|  | **F** | **df** | **P value** | **partial η^2^** |
| **HC (M-Subject)** | 0.01 | (1, 201) | 0.93 | 0.00 |
| **PS (Ex)** | 11.00 | (1,70) | 0.001* | 0.14 |
| **NS (Dm)** | 4.41 | (1, 82) | 0.04* | 0.05 |

Three-way (2 ’subject’ × 3 ‘group’ × 14 ‘temporal delay’) repeated-measures ANOVA on the yes-rate of SoA was conducted. All post hoc multiple comparisons were conducted using Shaffer’s modified sequentially rejective Bonferroni procedure, with a significance level of P < 0.05 (*). The Greenhouse-Geisser correction ε (=0.26) was used to evaluate F ratios for repeated measures involving more than one degree of freedom.

We found no significant main effect of subject, but significant main effects of group and temporal delay. There was also significant interactions between subject and group, subject and temporal delay, group and temporal delay, and between subject, group, and temporal delay. To investigate the significance of the interaction between subject and group, we performed simple main effects analyses. There was no significant simple main effect of HC (M-Subject), but significant simple main effect of PS (Ex) and of NS (Dm).

HC, healthy control; PS, paranoid-type schizophrenia; NS, negative symptom-predominant schizophrenia; Dm, diminished SoA.

**Supplementary Table 5. Statistical analysis of Sense of agency judgments in behavioral data.**

| **ANOVA TABLE** | | | | |
| --- | --- | --- | --- | --- |
|  | **F** | **df** | **P value** | **partial η^2^** |
| **Group** | 62.04 | (2, 78) | < .001* | 0.61 |
| **Temporal Delay** | 256.81 | (13, 1014) | < .001* | 0.77 |
| **Group x Temporal Delay** | 12.18 | (26, 1014) | < .001* | 0.24 |

| **Multiple comparison for Group** | | | | |
| --- | --- | --- | --- | --- |
|  | **t** | **df** | **P value** |  |
| **HC vs. PS** | 7.83 | 78 | < .001* | HC < PS |
| **HC vs. NS** | 4.02 | 78 | < .001* | HC > NS |
| **PS vs. NS** | 10.57 | 78 | < .001* | PS > NS |

| **Simple main effects of group within each temporal delay** | | | | | | | |  |
| --- | --- | --- | --- | --- | --- | --- | --- | --- |
|  | **F** | | **df** | | **P value** | | **partial η^2^** |  |
| **Temporal Delay (ms)** |  | |  | |  | |  |  |
| **EPA trial** |  | |  | |  | |  |  |
| **-100** | 8.06 | | (2, 78) | | < .001* | | 0.17 |  |
| **0** | 8.78 | | (2, 78) | | < .001* | | 0.18 |  |
| **100** | 6.09 | | (2, 78) | | < .05* | | 0.14 |  |
| **Action-linked trial** |  | | | | | |  |  |
| **0** | 5.49 | | (2, 78) | | < .05* | | 0.12 |  |
| **100** | 2.07 | | (2, 78) | | 0.13 | | 0.05 |  |
| **200** | 6.56 | | (2, 78) | | < .05* | | 0.14 |  |
| **300** | 21.20 | | (2, 78) | | < .001* | | 0.35 |  |
| **400** | 24.54 | | (2, 78) | | < .001* | | 0.39 |  |
| **500** | 32.88 | | (2, 78) | | < .001* | | 0.46 |  |
| **600** | 43.10 | | (2, 78) | | < .001* | | 0.53 |  |
| **700** | 46.60 | | (2, 78) | | < .001* | | 0.54 |  |
| **800** | 31.07 | | (2, 78) | | < .001* | | 0.44 |  |
| **900** | 24.69 | | (2, 78) | | < .001* | | 0.39 |  |
| **1000** | 18.75 | | (2, 78) | | < .001* | | 0.32 |  |
| **Multiple comparison for group within each temporal delay** | | | | | | | | |
|  | | **P value** | | **P value** | | **P value** | | |
| **Temporal Delay (ms)** | | **HC vs. PS** | | **HC vs. NS** | | **PS vs. NS** | | |
| **EPA trial** | |  | |  | |  | | |
| **-100** | | < .05* | | 0.88 | | < .05* | | |
| **0** | | < .001* | | 0.59 | | < .05* | | |
| **100** | | < .05* | | 0.59 | | < .05* | | |
| **Action-linked trial** | |  | | | | | | |
| **0** | | 0.13 | | < .05* | | 0.05 | | |
| **100** | | ns. | | ns. | | ns. | | |
| **200** | | 0.39 | | < .05* | | < .05* | | |
| **300** | | < .05* | | < .001* | | < .001* | | |
| **400** | | < .001* | | < .001* | | < .001* | | |
| **500** | | < .001* | | < .001* | | < .001* | | |
| **600** | | < .001* | | < .05* | | < .001* | | |
| **700** | | < .001* | | < .05* | | < .001* | | |
| **800** | | < .001* | | 0.31 | | < .001* | | |
| **900** | | < .001* | | 0.65 | | < .001* | | |
| **1000** | | < .001* | | 0.84 | | < .001* | | |

A two-way (3 ‘group’ × 14 ‘temporal delay’) repeated-measures ANOVA was conducted on the yes-rate of SoA. All post hoc multiple comparisons were performed using Shaffer’s modified sequentially rejective Bonferroni procedure with a significance level of P < 0.05 (*). The Greenhouse-Geisser correction ε (=0.33) was used to evaluate F ratios for repeated measures involving more than one degree of freedom.

A significant main effect of group and temporal delay, as well as a significant interaction between group and temporal delay, were found. Regarding the main effect of group, multiple comparisons revealed that the yes-rate for SoA was significantly higher in PS than in HC and NS, and significantly lower in NS than in HC. To investigate the significance of the interaction, simple main effects analyses were performed. A significant simple main effect of group was found at each temporal delay except for the 100 ms action-linked trial. Multiple comparisons for group at each temporal delay showed that the results were largely consistent with previous reports by Maeda et al. (2013).

HC, healthy control; PS, paranoid-type schizophrenia; NS, negative symptom-predominant schizophrenia; ns., at the 100 ms of the action-linked trial, no significant differences were observed at the stage of testing for simple main effect of group.
